# Uncommon Drug-Induced Hidradenitis Suppurativa: A Case Report of A Patient on Lithium Therapy

**DOI:** 10.7759/cureus.72049

**Published:** 2024-10-21

**Authors:** Tiffany Wut, Anastasiya Vynnytska, Aisha Ali, Frederick Tiesenga

**Affiliations:** 1 Medicine, St. George's University School of Medicine, St. George, GRD; 2 Surgery, St. George's University School of Medicine, St. George, GRD; 3 General Surgery, West Suburban Medical Center, Chicago, USA

**Keywords:** acne inversa, bipolar disorder, comorbidities, hidradenitis suppurativa, lithium, nodules, quality of life, schizophrenia

## Abstract

Hidradenitis suppurativa (HS) is not fully understood and is regarded as a multifactorial condition diagnosed based on clinical evaluation. Smoking, obesity, and hormonal imbalances may be the underlying associations of HS. The incidence of HS is greater in patients with diabetes. Psychiatric illnesses and their medications have been linked to new-onset and worsening of HS.

This case report discusses a 35-year-old Hispanic male, a non-smoker with a past medical history of schizophrenia and bipolar disorder, who was diagnosed with axillary HS after initiating lithium therapy. He experienced worsening HS and reached Hurley stage III, for which surgical excision was offered as the definitive treatment.

Here, we present a case report of HS after the initiation of lithium therapy, focusing on the underlying pathological process of this skin condition. Despite limited data on this topic, clinical features, management of the condition, and possible disease development prevention methods will be emphasized.

## Introduction

Hidradenitis suppurativa (HS) is a chronic, recurrent, multifactorial inflammatory skin condition known as acne inversa, defined by disordered terminal follicular pilosebaceous units [1]. There are numerous ways this can present, but usually, HS is characterized by subcutaneous tissues marked by deep, painful nodules and abscesses with draining sinus tracts [1]. It usually develops after puberty and is characterized by inflamed lesions in areas of the body with apocrine glands, most commonly in the underarms, groin, and anogenital regions [1,2]. HS symptoms are split into three categories by the Hurley staging system. Stage I presents with one or more nodules, which may or may not turn into an abscess without affecting sinus tracts or causing scarring. Stage II presents recurrent nodules and abscesses with sinus tract formation and/or scarring. Lastly, stage III is the most severe and consists of diffuse interconnected abscesses, sinus tracts, and scarring across the entire body [3].

The pathogenesis of HS is intricate and only partially understood. According to Agnese, autoinflammation is the primary driver of disease development. Genetics and the cutaneous microbiome play a role in developing chronic inflammation and lesion formation [3]. The direct cause of HS is not known, but some of the risk factors include smoking, metabolic syndrome, obesity, and diabetes. Literature suggests that 70% to 89% of the patients with HS are smokers, indicating that tobacco is a triggering factor for HS [2]. Obesity contributes to HS through multiple mechanisms, including sweat retention and disruption of hormonal balance. Skin-to-skin contact causes shearing, potentially triggering follicular plugging. Such contact can increase keratin hydration in sweat glands, narrowing follicular openings and causing pore blockage. Moreover, obesity alters hormonal metabolism, resulting in excess androgens, which can further exacerbate follicular plugging by coarsening hair shafts [1-3].

HS adversely impacts the quality of life in numerous ways [2]. Addressing the existing risk factors is essential in treating HS. Effective disease management involves lifestyle modifications alongside pharmacotherapy and procedures. Diagnosing HS relies on clinical criteria to differentiate it from other conditions like acne, folliculitis, and pilonidal cysts. While treatment remains challenging, many patients experience recurrent episodes and significant morbidity [4]. Various therapeutic methods for HS are available, including topical treatments, systemic antibiotics, hormonal therapies, biologic treatments, and surgery [4,5]. Despite these interventions, many patients experience recurrent episodes and significant morbidity.

Although much less documented, HS has been linked to certain medications, such as lithium [6]. Lithium increases neutrophil chemotaxis, causing lysosomal enzyme release, a process vital for follicular hyperkeratosis and the subsequent formation of acne [6,7]. This inflammatory response is similar to the pathological presentation of HS, suggesting a strong connection between lithium and HS. In this case report, our patient has schizophrenia and bipolar disorder and is currently on lithium. This inflammatory response is similar to the pathological presentation of HS, suggesting a strong connection between lithium and HS, although this association remains insufficiently studied [6,7]. This case report details an instance of HS, emphasizing specific diagnostic challenges and treatment outcomes, enhancing the broader understanding of this debilitating condition.

## Case presentation

A 35-year-old Hispanic male presented to an outpatient clinic for consultation of HS stage III to the right axilla. The patient reported experiencing frequent painful flare-ups and purulent drainage coming from the right axilla. His past medical history consists of HS on the left axilla, which started in 2014 and was surgically excised in 2017. In 2013, the patient was diagnosed with schizophrenia and manic episodes with depression, for which he was started on aripiprazole (30mg daily), lamotrigine (100 mg daily), and lithium (1200 mg daily). His medical history was negative for alcohol or illicit drug use. He is a lifelong non-smoker with no allergies. 

The patient presented with a body weight of 217 pounds, resulting in a body mass index of 31.1 kg/m^2^, which is classified as obese. His weight has increased from 180 pounds since the first HS diagnosis in 2014. The patient was observed to have cystic facial acne, including a beard, without swollen lymph nodes or any rashes. Physical examination showed 15x12x2.5cm necrotic, purulent, interconnected abscesses with significant scarring lesions extending through the full depth of the dermis. HS spread from the right axilla medially to the torso and laterally to the inner arm. 

**Figure 1:**
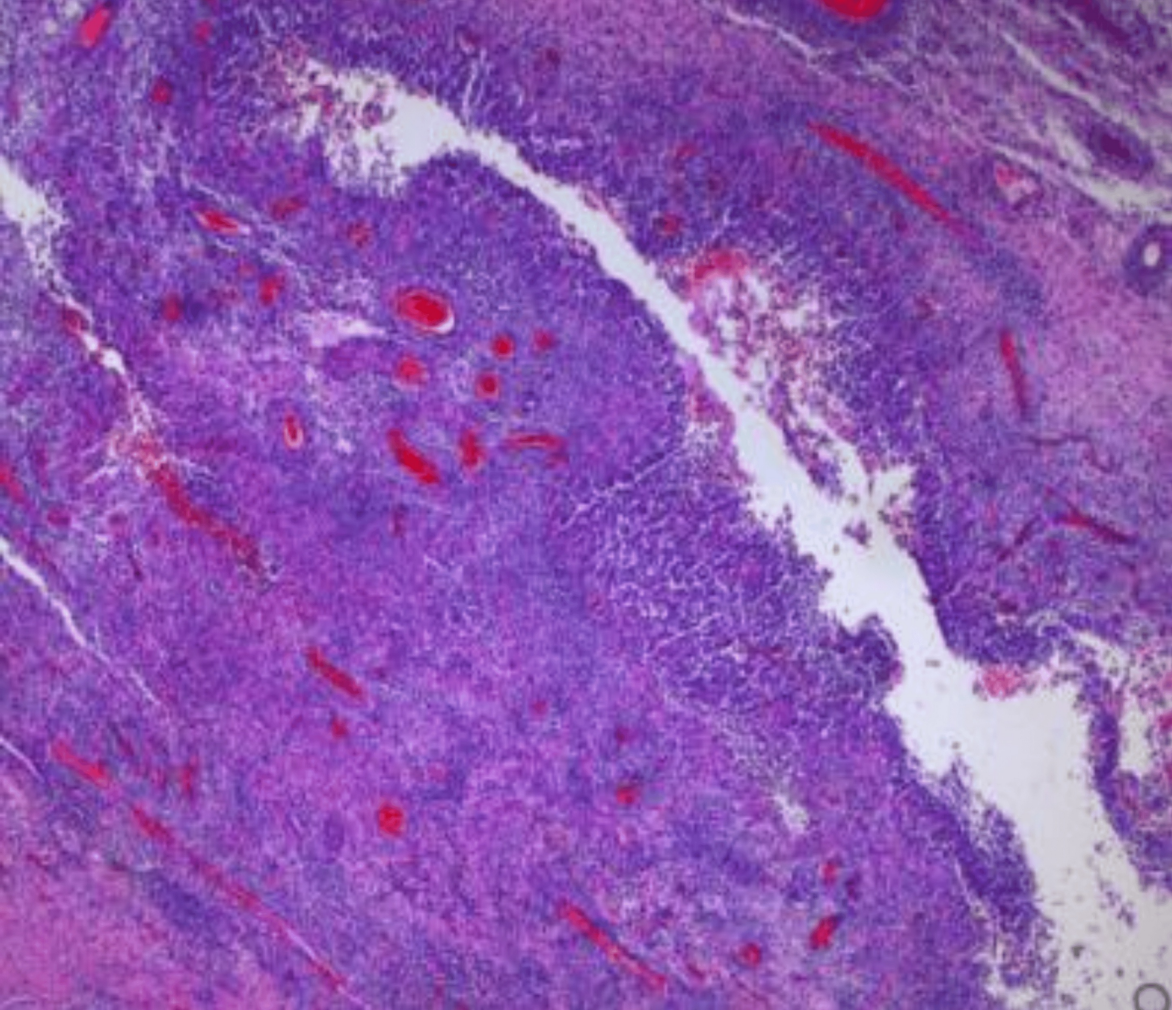
Pathology of excised right axillary tissue Hematoxylin and eosin stain

The patient underwent excision of right axillary HS, and the operating team sent 10.5x9.0x2.5cm tissue for pathological evaluation (Figure 1). The report showed skin with subcutaneous tissue with focal abscess, acute and chronic inflammation, and reactive changes. The results were negative for malignancy. These findings supported the diagnosis of HS Hurley stage III.

**Figure 2:**
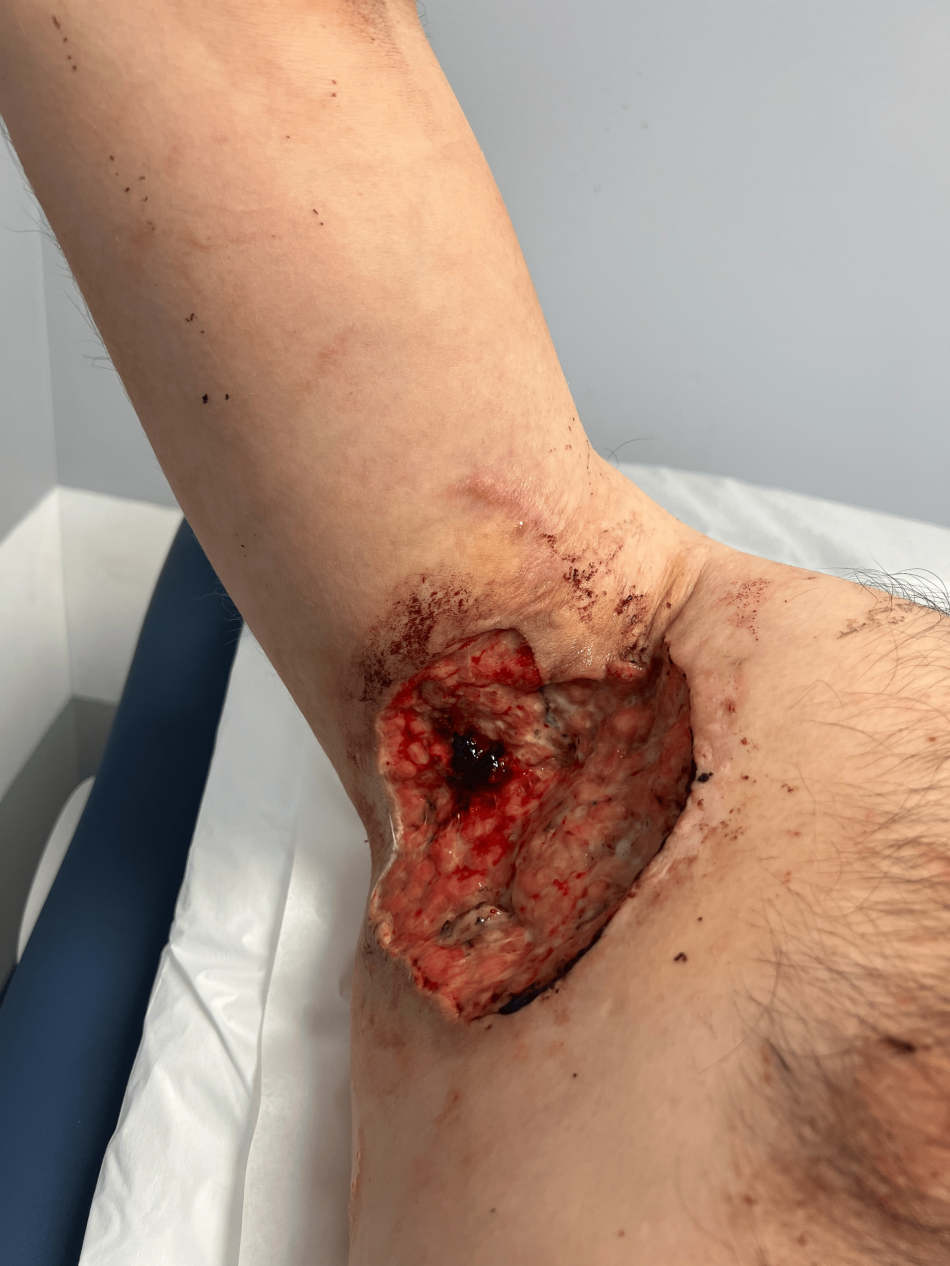
One week post-operative: Healing right axillary wound (15x12x2.5 cm)

Postoperatively, the patient was advised on a proper wet-to-dry dressing technique at home. He was also prescribed daily multivitamins with vitamin C and Zinc to promote healing. On physical examination, the right axillary wound presented with fibrous and granulation tissue, non-tender and without active bleeding (Figure 2).

One day post-operative, the patient was evaluated in the outpatient clinic, and the surgical dressing was removed. He was advised to clean his wound with saline and apply wet-to-dry dressing changes twice a day (BID)/three times a day (TID) at home. He was also advised to continue taking daily multivitamins with vitamin C and Zinc. The patient was scheduled for frequent follow-ups every week in the outpatient clinic. 

**Figure 3:**
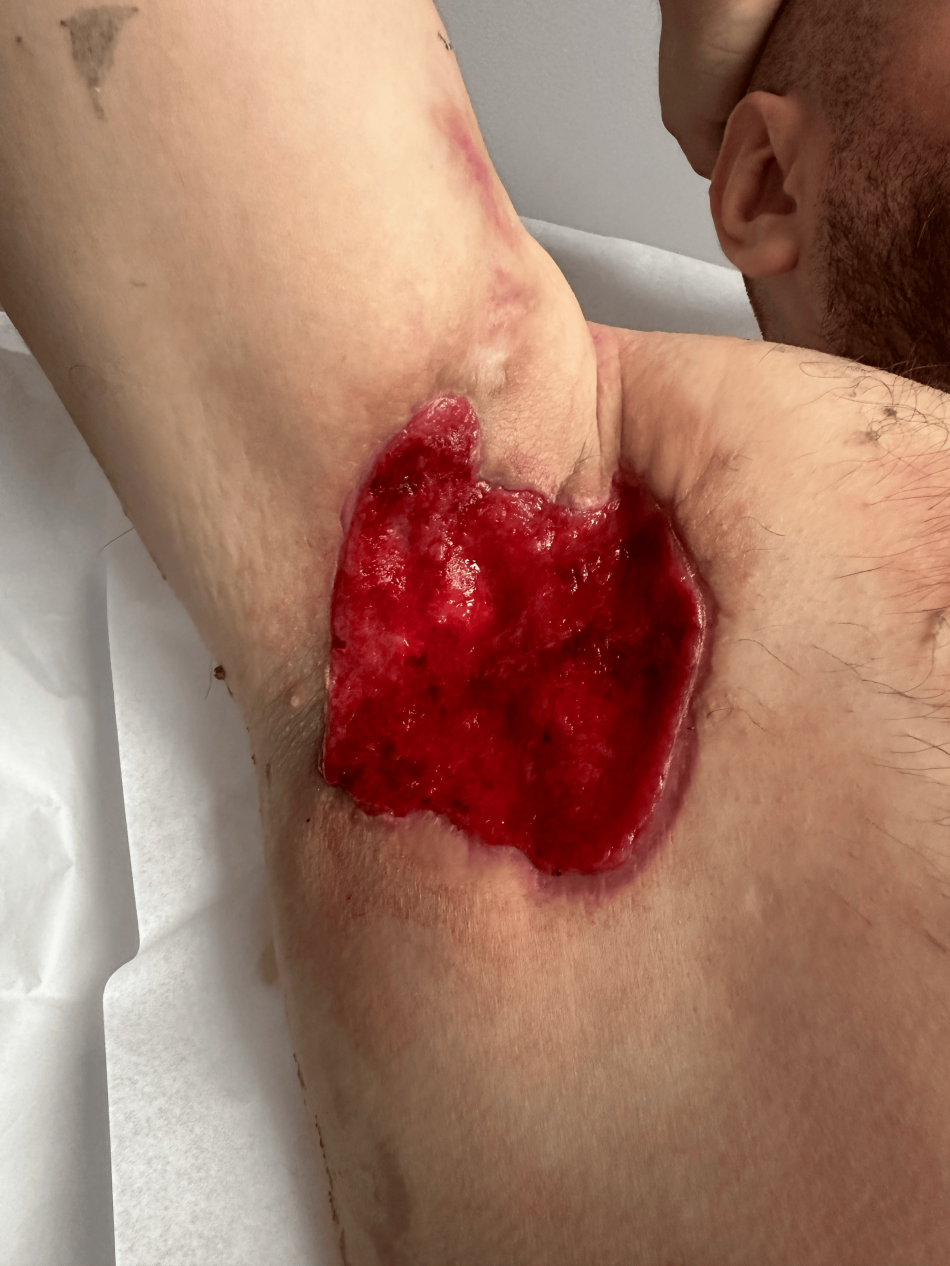
Three weeks post-operative: Healing right axillary wound (10x10x0.5 cm)

After two weeks, the wound care was switched to dry packing TID with the continuation of taking multivitamins. The right axillary wound was observed as moist, glossy, and with some slough. The size of the wound remained the same: 15x12x2.5 cm, non-tender, and with no active bleeding. Three weeks postoperatively, the wound decreased and was measured 10x10x0.5cm. It appeared to be properly healed with some hypergranulation tissue (Figure 3). Silver nitrate was applied to treat hypergranulation tissue, which the patient tolerated well. The patient will continue receiving outpatient treatment, focusing on complete wound healing. 

## Discussion

HS has an estimated global prevalence of up to 4%, with the highest rates found in the African American population (1.3%) and the lowest among Hispanics and Latinos (0.07%) [8,9]. Meta-analyses have shown that HS is more prevalent in females when compared to males [10]. There have also been significant studies underlying the risk factors for HS, such as smoking, metabolic syndrome, obesity, and diabetes, with smoking being particularly highlighted, as 70% to 89% of patients with HS are smokers [3]. HS also presents with other dermatologic comorbidities such as acne, dissecting cellulitis of the scalp, pilonidal disease, and pyoderma gangrenosum [11]. However, the etiology of HS remains unclear, and its prevalence needs more awareness.

There have been recorded high rates of comorbid psychological illnesses in HS patients as well [11]. Garg et al. study illustrates how psychiatric comorbidities are prevalent among HS patients, with depression being observed in up to 26% of HS patients [11]. A nationwide cohort study found that schizophrenia is four times more common in patients with HS. Furthermore, the prevalence of bipolar disorder is significantly higher in the HS population as well [12]. Authors propose there might be similar underlying immune system abnormalities present in HS and psychiatric disorders, such as depression, schizophrenia, and bipolar disorder. While immune system irregularities and environmental factors are great driving forces of HS illness, a commonly overlooked culprit is medications, which can either precipitate or worsen a patient's symptoms. This suggests the possibility that being diagnosed with a psychiatric disorder could present as a risk factor for developing HS. In this case report, this patient is obese and had HS in the contralateral armpit in the past, both of which increase his risk for future HS flares. However, the patient identifies as a Hispanic male and has never smoked, which places him in a demographic where HS is less common. One possible contributing factor to his recurrent HS could be his medical history of schizophrenia and bipolar disorder.

Studies have shown that medications used to treat psychiatric disorders, like lithium, can predispose individuals to or worsen symptoms of HS. Lithium is frequently used to treat bipolar disorder, but it carries a significant burden and a narrow therapeutic index, making patients vulnerable to lithium toxicity [13]. Adverse effects of lithium include but are not limited to weight gain, nephrotoxicity, and thyroid dysfunction. Notably, lithium is also associated with dermatologic conditions such as acne, psoriasis, alopecia, follicular inflammation, and maculopapular rashes [14]. Lithium increases neutrophil chemotaxis, causing lysosomal enzyme release, a process vital for follicular hyperkeratosis and subsequent formation or exacerbation of acne [7]. This inflammatory response is similar to the pathological presentation of HS, suggesting a strong connection between lithium and HS, although this association remains insufficiently studied.

The association between lithium and HS has been documented globally [15-17]. A retrospective study conducted by Benhadou et al. in 2020 further strengthened the association by documenting the new onset or worsening HS symptoms following the initiation of lithium therapy [18]. Data suggests the pathophysiology is similar to lithium-induced acne and psoriasis. Another case study by Chaudhari et al. offered compelling evidence of lithium-induced HS, documenting two cases where the initiation of lithium was followed by a new onset of HS symptoms within a month. Both patients had normal serum lithium ranges, and neither one felt any relief with doxycycline or topical applicants. Improvement in their skin condition only occurred after discontinuing lithium and switching to other medications [6].

This case highlights a Hispanic American male with schizophrenia and bipolar disorder who belongs to a demographic with the lowest HS prevalence yet required two surgical interventions in the past 10 years. The patient had been taking a high dose of lithium (1200 mg daily) for an extended period, further supporting the association between lithium and the severity of HS. Although every person reacts differently to illnesses and medications, the patients participating in the Chaudhari et al. study took 600 mg and 325 mg of lithium daily; these dosages were sufficient to initiate HS in just four weeks [6]. The patient, in this case, has been on a significantly higher dosage of lithium for an extended period, which may have triggered severe HS flare-ups. As a result, other medications were inadequate, and surgery was ultimately pursued as a last resort.

Lithium-induced cutaneous lesions can occur due to similar pathologic processes; a lower dose of lithium could have eliminated the patient's necessity to undergo the surgery. Priyadarshini et al. report patients treated with a lower lithium dosage and, thus, a lower serum lithium level had a reduced risk for cutaneous lesions [19]. Lithium was able to stabilize patient mood even at a lower serum concentration (<0.8mEq/L). Furthermore, a survival analysis after 10 years demonstrated patients with higher dosages and serum levels of lithium were linked to an increased prevalence of skin lesions. This finding can be extrapolated to lithium-induced HS; in patients who can not tolerate the discontinuation of lithium, reducing the lithium dosage might have mitigated the severity of HS symptoms [19].

Patients affected by HS often experience a lower quality of life, along with significantly higher rates of anxiety, depression, and suicide [11]. This patient exemplifies these statistics, having been hospitalized twice due to HS and suffering from comorbid depression. The severity of his condition places him at heightened risk for a diminished quality of life, extreme functional impairment, and even suicide. Considering the significant risks associated with HS, continued research into its risk factors and causes is crucial. Increased awareness and research on lithium-induced HS might have led to this patient being prescribed a lower dose or an alternative treatment for his bipolar disorder, potentially preventing the severity of his symptoms and the need for a second surgical excision.

## Conclusions

Although the risk factors for HS, such as environmental influences, genetics, ethnicity, and immune system irregularities - including smoking, diabetes, obesity, and metabolic syndrome - are only partly understood, this case study highlights a potential association between HS and psychological illnesses, particularly in relation to medications like lithium. Lithium and HS share strong similarities in pathogenesis, which explains higher rates of comorbidities of HS in psychiatric patients.

Clinicians must be aware of the risks of HS development in patients with psychiatric conditions to prevent the disease or provide early care to avoid surgical interventions. Although surgical intervention demonstrates a successful rate of treatment and significantly improves the patient's quality of life, clinicians can focus on monitoring lithium levels closely, suggesting lifestyle modifications, obtaining a history of patients' past skin conditions, and advising them to report any new skin changes upon starting lithium. These recommendations could help detect new-onset HS and prevent its progression to stage III. Further research is needed to better understand the prevalence of HS in patients with psychiatric disorders who are undergoing lithium therapy at various therapeutic ranges, as well as the associated timelines for HS stage progression.
